# Molecular detection of *Ehrlichia canis* in dogs from three districts in Punjab (Pakistan)

**DOI:** 10.1002/vms3.94

**Published:** 2018-02-07

**Authors:** Muhammad I. Malik, Muhammad Qamar, Quratul Ain, Malik F. Hussain, Mustapha Dahmani, Mazhar Ayaz, Asim K. Mahmood, Bernard Davoust, Rehan S. Shaikh, Furhan Iqbal

**Affiliations:** ^1^ Institute of Molecular Biology and Biotechnology Bahauddin Zakariya University Multan Multan Pakistan; ^2^ Department of Zoology PMAS Arid and Agriculture University Rawalpindi Rawalpindi Pakistan; ^3^ Institute of Pure and Applied Biology Zoology Division Bahauddin Zakariya University Multan Pakistan; ^4^ Research Unit of Emerging Infectious and Tropical Diseases (URMITE) UMR CNRS 7278 IRD 198 Aix‐Marseille‐University Marseille France; ^5^ Faculty of Veterinary Sciences Bahauddin Zakariya University Multan Pakistan; ^6^ Pet centre University of Veterinary and Animal Sciences Lahore Pakistan

**Keywords:** *Ehrlichia canis*, 16S rRNA gene, PCR, Dog breeds, Haematology

## Abstract

Canine monocytic ehrlichiosis is a tick‐borne disease caused by an intracellular alpha‐proteobacterium, *Ehrlichia canis,* which replicates within mononuclear cells in the host. This study was designed to use a polymerase chain reaction (PCR) protocol for the molecular detection of *E. canis* by the amplification of a portion of its 16S rRNA gene, as well as the effects of this alpha‐proteobacterium on the haematological parameters of the sampled dogs and the risk factors associated with *E. canis* infection. A total of 151 blood samples were collected from dogs of various breeds at three sampling sites (Lahore, Rawalpindi/Islamabad and Multan) in Punjab, Pakistan. Data regarding the epidemiological factors (including age, gender, breed, body temperature, deworming, vaccination, mucous membrane status, hydration status, the presence of haematuria and tick infestation) were collected through a questionnaire at the time of sample collection. A 400 bp DNA fragment of the 16S rRNA gene of *E. canis* was amplified from 42 dog blood samples (28% of the total), [Lahore (*N* = 24), Rawalpindi/Islamabad (*N* = 13) and Multan (*N* = 05)] through PCR. Data analysis revealed that the character of the animals (age, sex and breed) had no significant association (*P* > 0.05) with the presence of *E. canis*. Various haematological parameters were also compared, and the results revealed that all of the parameters remained unaffected, except significantly lower white blood cell counts (*P* = 0.004) in *E. canis*‐positive blood samples, as compared with the control group. We concluded that this is the first molecular confirmation of canine infection by *E. canis* using PCR. Moreover, no specific epidemiological parameter was found associated with the prevalence of *E. canis* in dogs.

## Introduction

Dogs are among the most common pets, and as their population has increased tremendously, parasitic diseases are a major health concern (McBride *et al*. 1996). Among these parasitic and infectious diseases, canine monocytic ehrlichiosis is a tick‐borne disease caused by an obligate intracellular alpha‐proteobacterium, *Ehrlichia canis* (*E. canis*), which replicates within mononuclear cells in the host (Harrus & Waner 2011). Canine ehrlichiosis has been reported from subtropical and tropical areas of the world where it is one of the very common disease reported during veterinary practice in dogs (Rani *et al*. 2011). One of the major reasons for the common occurrence of *E. canis* is the tick vector, *Rhipicephalus sanguineus* sensu lato*,* which is common in rural and urban areas in tropical and subtropical areas of the world (Aguiar *et al*. 2007; Dantas‐Torres 2008; Aktas 2014; Aktas *et al*. 2015).

Three consecutive phases of canine ehrlichiosis have been reported in the literature; the subclinical, acute and chronic phase. Thrombocytopenia, variable leucopenia and anaemia are commonly found to be associated with ehrlichiosis (Greene 2013). The acute phase of this disease is characterized by symptoms of fever, depression, dyspnoea, anorexia, lymphadenopathy and slight weight loss (Iqbal *et al*. 1994). In the chronic disease, dogs have been reported suffering from haemorrhages, epistaxis, peripheral oedema, emaciation and hypotensive shock leading to death (Ristic & Woldehiwet 1993). To our knowledge, no information is available in literature regarding the prevalence of *E. canis* in dogs in Pakistan, the present study aimed to perform a molecular detection of canine infection by *E. canis* in Pakistan. As this pathogen is found in the blood, we are also reporting the comparison of various haematological parameters between *E. canis‐*positive and ‐negative animals.

## Materials and methods

### Sample and data collection

Blood samples from 151 dogs were randomly collected from pet clinics in Lahore (*N* = 50); Rawalpindi/Islamabad (*N* = 50) and Multan (*N* = 51), districts of Punjab, during June–December 2014 All of the dogs included in the study were pets, except for stray dogs (*N* = 2), and informed consent was obtained from pet owners before enrolling them in the study. Sampled dogs included healthy animals as well as those having clinical symptoms including fever (diagnosed with thermometer) pale mucus membrane, haematuria and vomiting (physical examination). Blood samples were collected from the saphenous vein of the animals and immediately preserved in EDTA tubes. A questionnaire was filled at the sampling site in order to gather data (including age, sex, breed, body temperature, deworming, vaccination, mucous membrane status, hydration status, the presence of haematuria and tick infestation) and to report risk factor(s) associated with the prevalence of *E*. *canis* DNA in dogs, if any *(de Castro et al*. 2004; Shipov *et al*. 2008). The body of each dog, with special attention to the ears, was examined for the presence of ticks. If present, they were removed with forceps and placed in bottles with moistened cotton wool and were then transferred to the laboratory for identification using taxonomic keys (Aktas 2014).

### DNA extraction

The inorganic method of DNA extraction was used, following Shahnawaz *et al*. (2011). The quality of the DNA extracted was assessed with optical density counts at 260/280 nm and submerged gel electrophoresis to determine its purity and integrity.

### PCR amplification

A set of previously reported oligonucleotide primers, Fwd ECA: 5′‐AAC ACA TGC AAG TCG AAC GGA‐3 and Rev HE3: 5′‐TAT AGG TAC CGT CAT TAT CTT CCC TAT‐3, was used to amplify the 16S rRNA gene sequences of *E. canis,* as previously reported (Wen *et al*. 1997). *E. canis*‐positive samples, kindly donated by Prof. Dr. Diego Fernando Eiras from National University of La Plata Facultad de Ciencias Veterinarias Argentina, and negative controls (polymerase chain reaction (PCR) mixture without DNA) were amplified during each PCR as positive and negative controls, respectively.

### Haematological analysis

Various haematological parameters in blood samples from dogs, i.e. white blood cells, platelets, red blood cells, haemoglobin, mean corpuscular volume and packed cell volume, were determined in blood samples from *E. canis‐*positive and ‐negative dogs by an automated haematology analyser (Abbott Cell‐Dyn 3700), Illiois USA).

### Statistical analysis

All of data are expressed as mean ± standard deviation. Minitab Statistical package (Pennsylvania, USA) was used for the statistical analysis of the results. Animals were divided into two age groups, animals up to 1 year (juvenile) and more than 1 year (mature). The association between the presence of *E. canis* and the various risk factors, e.g. the sex and age of the animal, was evaluated by contingency table analysis using the Fisher's exact test (for 2 x 2 tables). Two sample *t*‐tests were calculated to compare the various studies haematological parameters between *E. canis*‐positive and ‐negative blood samples.

## Results

All dogs were examined for the presence of ixodid ticks. Of the 151 dogs, 11 (7.3%) were infested with adult and nymphal *Rhipicephalus sanguineus s.l*. Forty‐eight ticks (29 nymphs and 19 adults) were removed from dogs.

PCR amplification revealed that 42 out of 151 (28%) blood samples were positive for *E. canis,* as they amplified a 400‐bp amplicon using the set of primers targeting the 16S rRNA gene of *E. canis*. The prevalence of *E. canis* varied significantly (*P* < 0.001) between the three sampling sites. Maximum prevalence of *E. canis* was observed in the Lahore district (48%) followed by Rawalpindi/Islamabad (26%) and Multan (10%) (Table 1).

**Table 1:** Prevalence of *Ehrlichia canis* as detected by polymerase chain reaction (PCR) during this study in blood samples of dogs collected from three selected sampling sites (Lahore, Rawalpindi/Islamabad and Multan) in Punjab Province

Sampling sites	Total samples	*Ehrlichia canis* PCR positive	*Ehrlichia canis* PCR negative	*P*‐valve
Lahore	50	24 (48%)	26 (52%)	*P *<* *0.001***
Islamabad/Rawalpindi	50	13 (26%)	37 (74%)	
Multan	51	05 (10%)	46 (90%)	
Grand total	151	42 (28%)	109 (72%)	

*P*‐values represents the results of one‐way ANOVA calculated for *Ehrlichia Canis* prevalence in three cities. *P* < 0.001 =  Highly significant (***).

The one‐way ANOVA was calculated to report the prevalence of parasite in various dog breeds enrolled in present study and the results indicated that prevalence of *E. canis* was not restricted (*P* > 0.05) to a particular breed. The highest prevalence of *E. canis* was detected in stray dogs (100%; *N* = 2) while the lowest was observed in Labradors (15.38%; *N* = 26). *E. canis* was not detected in Shih Tzu, Spaniel, Pug and Rottweiler breeds during this study (Table 2). Analysis of data, collected through a questionnaire at the sampling site, revealed that none of the studied epidemiological parameters were found associated (*P* > 0.05) with the prevalence of *E. canis* in dogs (Table 3).

**Table 2:** Breed‐wise prevalence of *Ehrlichia canis* in blood samples of dogs collected from three selected sampling sites (Lahore, Rawalpindi/Islamabad and Multan) in Punjab during this study

Breed	Number of samples	*Ehrlichia canis* positive	Prevalence (%)	*Ehrlichia canis* negative	Prevalence (%)
German Shepherd	39	11	28	28	72
Boxer	04	02	50	02	50
Labrador	26	04	15.4	22	84.6
Stray	02	02	100	00	00
Cross	31	09	29	22	71
Bully	26	09	35	17	75
Pug	3	00	00	03	100
Russian	6	01	17	05	83
Shidzoo	01	00	00	01	100
Pointer	05	03	60	02	40
Spanial	03	00	00	03	100

*P*‐values show the results of one‐way ANOVA. *P* > 0.05 =  Non‐significant.

**Table 3:** Association of gender and age with prevalence of *Ehrlichia canis* in blood samples of dog collected from three sampling sites (Lahore, Rawalpindi/Islamabad and Multan) in Punjab during this study

Parameters	Total samples	*Ehrlichia canis*‐positive samples	*Ehrlichia canis*‐negative samples	*P*‐value
Sex				
Male	82	27 (32.9%)	55 (67.07%)	0.15
Female	69	15 (21.7%)	54 (78.2%)	
Age				
>1 Year	127	33 (25.98%)	94 (74.01%)	0.32
<1 Year	24	09 (37.5%)	15 (62.5%)	
Ticks on dogs				
Present	11	3 (27%)	8 (73%)	1
Absent	140	39 (28%)	101 (72%)	
Body temperature				
Normal	118	32 (27%)	86 (73%)	0.8
Fever	33	10 (30%)	23 (70%)	
Mucus membrane				
Normal	122	33 (27%)	89 (73%)	0.7
Pale	29	9 (31%)	20 (69%)	
Haematuria				
Present	25	7 (28%)	18 (72%)	1
Absent	126	34 (27%)	92 (73%)	
Vomiting				
Present	39	14 (36%)	25 (64%)	0.2
Absent	112	28 (25%)	84 (75%)	

Prevalence of the parasite is given in parenthesis. *P*‐value represents the results of the Fisher's exact test calculated for each studied parameter. *P* > 0.05 =  Non‐significant.

Comparison of the haematological parameters between *E. canis*‐positive (*N* = 42) and ‐negative blood (*N* = 109) samples from Lahore, Rawalpindi/Islamabad and Multan revealed that most of the studied parameters varied non‐significantly (*P* > 0.05) when compared between the two groups except white blood cell count that was significantly lower (*P* = 0.004) in *E. canis* positive than in blood samples where parasite was not detected (Table S1).

## Discussion

Canine monocytic ehrlichiosis is a tick‐borne disease of increasing importance in dogs, and has become an active area of research in recent years, although it remained unexplored in Pakistan. To our knowledge, this is the first report regarding the prevalence of *E. canis* DNA in dogs in Pakistan.

*E. canis* has been detected and reported in dogs from many parts of the world (Harrus *et al*. 2011; Rani *et al*. 2011; Sasaki *et al*. 2012; Ybañez *et al*. 2012; Nazari *et al*. 2013; Aktas *et al*. 2015; Inpankaew *et al*. 2016). An analysis of our results revealed a 28% overall prevalence of *E. canis* (*N* = 42) in the studied blood samples from the three districts, suggesting that *E. canis* is prevalent in Punjab province (Table 1) but the prevalence of *E. canis* varied significantly between three sampling areas indicating that geographical features and climatic conditions do affect the parasite prevalence (Rani *et al*. 2011; Mircean *et al*. 2012). In Turkey, a large study was conducted in the coastal provinces (Sakarya, Kocaeli, Mersin, Giresun and İzmir) as well as in land provinces (Elazig, Diyarbakır, Erzurum, Ankara, Nevşehir) in 2015 and it was observed through PCR and reverse line blotting (RLB) assays that 37/757 (4.9%) dogs were positive for *E. canis* (Aktas *et al*. 2015). In a similar study conducted in four different regions of India by using PCR technique, it was reported that 21% of the enrolled dogs were having *E. canis* in their blood (Rani *et al*. 2011). In a recent study conducted in St. Kitts (West Indies), the prevalence of *E. canis* in collected dog samples was found to be 27% using quantitative PCR (Kelly *et al*. 2013). Rojas *et al*. (2014) have also reported 34% prevalence of *E. canis* in dogs from Costa Rica (Rojas *et al*. 2014). An analysis of our results revealed a 28% overall prevalence of *E. canis* (*N* = 42) in the studied blood samples from the three districts, suggesting that *E. canis* is prevalent in Punjab province (Table 1). These sampling sites have different geographical and climatic conditions. Islamabad is located at 33.43°N 73.04°E at the northern edge of the Pothohar Plateau at the foot of the Margalla Hills. The city has a humid subtropical climate, with five seasons: winter (November–February), spring (March and April), summer (May and June), monsoon (July and August) and autumn (September and October). Lahore is located at 31°15′‐31°45′ N and 74°01′‐74°39′ E and has a semi‐arid climate. The hottest month is June, with high temperatures routinely exceeding 40°C. Multan (30°11' 44” N, 71°28' 31” E) has an arid climate with hot summers and mild winters. The city experiences some of the most extreme weather in the country. Our results indicated that prevalence of *E. canis* varied significantly (*P* < 0.001) between three sampling site that can be attributed to different climatic conditions that effects the prevalence of vector ticks and hence parasite prevalence. These variation in the prevalence of *E. canis* among the various studies discussed here could be due to many factors including the distribution and population density of the vector (Otranto *et al*. 2011), the sampling methodology and the characteristics of the targeted dog population (Gomes *et al*. 2010; De Miranda *et al*. 2014).

An analysis of risk factors indicated that male dogs were more often infected with *E. canis* than the females, but this association was statistically non‐significant (*P* = 0.15) confirming previous studies reporting that there is no correlation between the sex and the presence of *E. canis* infection in dogs (Rani *et al*. 2011; Aktas *et al*. 2015). Similarly, data regarding the age of the animals indicated that animals less than 1‐year‐old were more prone to *E. canis* infection compared with animals older than 1 year old, but this association was also statistically non‐significant (*P* > 0.05) indicating that neither sex nor the specific stage of life made subjects more prone to *E. canis* infection. This observation is contradictory to the results of a study conducted in Mexico, as they had reported higher incidence of *E. canis* infection in dogs of an age range between 2 and 4 years (Rodriguez‐Vivas *et al*. 2005).

The vectors of *E. canis* are reported to be *R. sanguineus* s.l. world wide. In addition, *Haemaphysalis longicornis* has been documented to be infected *by E. canis* in Korea (Kang *et al*. 2013). In this study, *R. sanguineus* s.l. ticks were collected and identified from 11 dogs in the three cities in Punjab. This observation confirms the previous findings that urban dogs are susceptible to *R. sanguineus* s.l. infestation (Melo *et al*. 2011; Rani *et al*. 2011). However, we did not find any correlation between presence of *E. canis* DNA with the presence of *R. sanguineus* s.l. on dogs (*P *=* *1). This finding is not consistent with a previous report from Turkey, in which *E. canis* infection in dogs was positively correlated with the presence of ticks (Aktas *et al*. 2013). Most of the animals sampled in this study were pets and groomed by the owners and ticks were only found on 11 of 151 (7.2%) sampled dogs. Out of these 11 dogs having tick burden, only 3 were found infected with *E. canis*. Good care and management of dogs by owners could be the reason that most dogs were not found to be infested with ticks, and hence, we have not seen a positive correlation between the presence of ticks and prevalence of *E. canis* in the blood of enrolled dogs. For 39 dogs in which *E. canis* were detected but ticks were not found on their bodies, we assume that dogs may had came across ticks during their daily routines (for example while on a walk in grassy parks) but ticks were removed by their owners during routine washing and checkup or ticks may also detach spontaneously after blood meal.

We have also tried to correlate various clinical signs (including body temperature, mucous membrane status, hydration status and the presence of haematuria) observed in sampled dogs with the prevalence of *E. canis,* but our results indicated that none of them was found to be associated with the presence of *E. canis* in dog's blood (*P* > 0.05 for all parameters) (Table 3). Our results are in overall agreement with a recent report from India in which it has been documented that none of the studied epidemiological factors was found to be associated with the presence of *E. canis* in dogs (Rani *et al*. 2011).

Diagnosis of ehrlichiosis can be challenging due to its different phases and multiple clinical manifestations. Canine monocytic ehrlichiosis should be suspected when a compatible history (living in or travelling to an endemic region, previous tick exposure), typical clinical signs and characteristic haematological and biochemical abnormalities are present (Harrus & Waner 2011). As ehrlichiosis is a vector‐borne disease, various haematological parameters were also compared between parasite‐positive (*N* = 42) and ‐negative (*N* = 109) blood samples. Our results indicated that white blood cell counts were significantly increased (*P* = 0.004) in dogs where *E. canis* was detected in blood than *E. canis*‐negative dogs (Table S1). Our results are in agreement with Shipov *et al*. (2008), as they had reported a higher number for WBC in dogs surviving with an *E. canis* infection (Shipov *et al*. 2008). Characteristic features of ehrlichiosis, like thrombocytopenia and pancytopenia, were not observed during present study. The potential reason for this could be the co‐infection of multiple parasites masking the typical symptoms of a particular disease. Often, subclinical infection with *E. canis* will go unrecognized, as most dogs are thought to control the infection immunologically, and infected dogs appear healthy until late in the infection, when pancytopenia, uveitis, weight loss and haemorrhagic disorders arise, and a diagnosis of ehrlichiosis is made (Gaunt *et al*. 2010; Greene 2013).

In conclusion, we have used an already established PCR protocol for the detection of *E. canis* in apparently healthy dog blood samples and this is first report from Pakistan on this topic. We are recommending the use of this PCR based protocol to veterinary practitioners and pet owners for the detection and/or confirmation of *E. canis* infection in dogs to improve their health status.

## Source of funding

The authors confirm that there was no specific research grant for this project to disclose.

## Conflict of interest

The authors declare no conflict of interest of any sort with anyone.

## Ethical statement

The authors confirm that the ethical policies of the journal, as noted on the journal's author guidelines page, have been adhered to and the appropriate ethical review committee approval has been received. All of the animal handling procedures and lab protocols were approved by the Ethics Committee of Institute of Molecular Biology and Biotechnology, Bahauddin Zakariya University Multan, Pakistan (Application No. IMBB/2014/MP84).

## Contributions

RSS designed the study; AKM, QUA and MFH collected the samples; MIM and MQ conducted the lab experiments; MA identifies the ticks collected from dogs; FI analysed the data; MD and BD prepared the manuscript.

## Supporting information


**Table S1.** Comparison of investigated haematological parameters between *Ehrlichia canis*‐positive (*N* = 42) and ‐negative (*N* = 109) blood samples of dogs (based on polymerase chain reaction results) collected from three sampling sites (Lahore, Rawalpindi/Islamabad and Multan) in Punjab. Data are presented as mean ± Standard deviation. *P*‐value represents the results of two sample test calculated for each parameter.
