# Supplementary material for: Molecular detection of Ehrlichia canis in dogs from three districts in Punjab (Pakistan)
Source: Vet Med Sci. 2018 Feb 7;4(2):126–32. doi: 10.1002/vms3.94 (PMC5979635; doi:10.1002/vms3.94)
Supplement: Supplementary file 1 — Table S1. Comparison of investigated haematological parameters between Ehrlichia canis‐positive (N = 42) and ‐negative (N = 109) blood samples of dogs (based on polymerase chain reaction results) collected from three sampling sites (Lahore, Rawalpindi/Islamabad and Multan) in Punjab. Data are presented as mean ± Standard deviation. P‐value represents the results of two sample test calculated for each parameter. [file VMS3-4-126-s001.doc]

Supplementary Table 1 Comparison of investigated hematological parameters between *Ehrlichia canis* positive (N = 42) and negative (N = 109) blood samples of dogs (basen on Polymerase Chain Reaction results) collected from three sampling sites (Lahore, Rawalpindi/Islamabad and Multan) in Punjab. Data is presented as mean ± Standard deviation. P –value represents the results of two sample test calculated for each parameter.

| Studied Parameters | Normal Range | *Ehrlichia canis* PCR negative dogs | *Ehrlichia canis* PCR positive dogs | P-Value |
| --- | --- | --- | --- | --- |
|  |
| WBC (10^3^/µL) | 6-17 | 15.47 ± 0.97 | 19.27 ± 0.84 | 0.004 ** |
| LYM (10^3^/µL) | 1-4.80 | 2.71 ± 0.32 | 3.06 ± 0.32 | 0.447 |
| GRA (10^3^/µL) | 3-12 | 12.03 ± 0.89 | 14.22 ± 0.77 | 0.066 |
| LY (%) | 12-30. | 18.5 ± 2.2 | 18.0 ± 1.4 | 0.849 |
| GR (%) | 62-87 | 77.6 ± 2.3 | 77.4 ± 1.5 | 0.941 |
| RBC (10^6^/µL) | 5.5-8.5 | 5.98 ± 0.26 | 5.92 ± 0.20 | 0.875 |
| HGB (g/dl) | 12-18 | 14.06 ± 0.64 | 14.28 ± 0.54 | 0.79 |
| HCT (%) | 37-55 | 38.4 ± 1.8 | 36.2 ± 1.3 | 0.311 |
| MCV (fl) | 60-77 | 67.28 ± 1.1 | 65.98 ± 0.64 | 0.297 |
| MCH (pg) | 19.5-24.5 | 23.66 ± 0.28 | 23.50 ± 0.17 | 0.625 |
| MCHC (g/dl) | 31-34 | 35.58 ± 0.44 | 35.94 ± 0.23 | 0.472 |
| PLT (10^3^/µL) | 200-500 | 284 ± 38 | 273 ± 26 | 0.802 |
| MPV (fl) | 3.9-11.1 | 8.06 ± 0.29 | 7.76 ± 0.17 | 0.378 |

P > 0.05 = Non significant P <0.01** = Significant

Where WBCs = White blood cells, LYM = Lymphocyte, GRA = Granulocytes, LY% =Relative (%) Content of Lymphocytes, GR% = Relative (%) Contentof Granulocytes, RBC = Red Blood Cells, HGB = Hemoglobin, HCT = Hematocrite, MCV = Mean volume of Erythrocytes in cubic micrometers, MCH = Mean Content of Hemoglobin, MCHC = Mean Concentration of Hemoglobin in Erythrocytes, PLT = Platelets, MPV = Mean Platelet Volume.
